# Has Gavi lived up to its promise? Quasi-experimental evidence on country immunisation rates and child mortality

**DOI:** 10.1136/bmjgh-2019-001789

**Published:** 2019-12-03

**Authors:** Pascal Jaupart, Lizzie Dipple, Stefan Dercon

**Affiliations:** 1 Centre for the Study of African Economies, University of Oxford, Oxford, UK; 2 Blavatnik School of Government, University of Oxford, Oxford, UK; 3 Department of Economics, University of Oxford, Oxford, UK

**Keywords:** vaccines, immunisation, child health, other study design, health policy

## Abstract

**Introduction:**

Gavi, the Vaccine Alliance, was set up in 2000 to improve access to vaccines for children living in the poorest countries. Funding has increased significantly over time, with Gavi disbursements reaching US $1.58 billion in 2015. We assess whether Gavi’s funding programmes have indeed increased immunisation coverage in 51 recipient countries for two key vaccines for 12–23 month olds: combined diphtheria, pertussis and tetanus (DPT) and measles. Additionally, we look at effects on infant and child mortality.

**Methods:**

Taking a difference-in-differences quasi-experimental approach to observational data, we estimate the impact of Gavi eligibility on immunisation coverage and mortality rates over time, using WHO/UNICEF figures covering 1995–2016. We control for economy size and population of each country as well as running a suite of robustness checks and sensitivity tests.

**Results:**

We find large and significant positive effects from Gavi’s funding programmes: on average a 12.02 percentage point increase in DPT immunisation coverage (95% CI 6.56 to 17.49) and an 8.81 percentage point increase in measles immunisation coverage (95% CI 3.58 to 14.04) over the period to 2016. Our estimates show Gavi support also induced 6.22 fewer infant deaths (95% CI −10.47 to −1.97) and 12.23 fewer under-five deaths (95% CI −19.66 to −4.79) per 1000 live births.

**Conclusion:**

Our findings provide evidence that Gavi has had a substantial impact on the fight against communicable diseases for improved population and child health in lower-income countries. In this case, the health policy to verticalise aid—specifically development assistance for health—via a specialised global fund has had positive outcomes.

**Summary**

**What is already known?**

- To date, only two previous independent studies have undertaken causal analyses of the effect of the introduction of Gavi, the Vaccine Alliance, on immunisation coverage.
- In an early study from 2006, Lu and colleagues found positive impacts of Gavi funding on diphtheria, pertussis and tetanus (DPT) immunisation rates, but their estimates were produced from only 10 years of data (1995–2004) and the statistical methods they used have been superseded.
- A more recent study by Dykstra and colleagues using a methodology restricted to less-poor countries and with lower statistical power found mainly null or insignificant impacts on access to vaccines thanks to Gavi support.

**What are the new findings?**

- Unlike the recent study, our positive findings for DPT and measles immunisation coverage back up earlier evidence of Gavi’s positive impact for its client countries, now using 15 years of data since Gavi’s inception and including the poorest countries, where we might expect to see large impacts.
- In addition, we find a significant reduction in infant and under-five mortality rates.
- We show that our findings survive an extensive battery of sensitivity checks.

**What do the new findings imply?**

- In this particular case, the creation of a specialised, immunisation-focused global fund has improved child health outcomes.
- Given large positive effects from the introduction of Gavi assistance, the transition process for countries graduating out of Gavi funding eligibility should be carefully managed, and studied as further data becomes available.

## Introduction

As well as seeing significant growth in the amount of international development assistance, the 2000s was a time of increased aid specialisation, particularly for health. This verticalisation movement was in part driven by the thematic focus of the Millennium Development Goals.^1 2^ Donor countries also wanted more control, and increasingly funnelled development financing through earmarked programmes or via specialised funds.^3 4^ Recent reviews of the evidence into this aid fragmentation have found both positive and negative results.^5–7^ Now that development assistance for health (DAH) is plateauing and with fund replenishments coming up, the efficacy of these specialised vertical health funds needs to be re-evaluated.^8^

One such initiative, Gavi, the Vaccine Alliance (previously the Global Alliance for Vaccines and Immunisation), was created in 2000 in response to stagnated immunisation coverage rates observed in the 1990s in developing countries. It is a global public–private partnership committed to improving access to vaccines for children living in the world’s poorest countries. Gavi’s strategy is the supply of new and underused vaccines and the strengthening of health and immunisation systems. The scale of Gavi’s operations has grown over time and, since 2013, disbursements have levelled off at just under US$1.50 billion per year on average.^9^ Gavi accounted for 5.11% of total global DAH in 2015.^8^ According to Gavi’s own figures, since the fund started it has reached 640 million children in low-income and middle-income countries, saving 9 million lives.^10^

Immunisation is seen as a key driver of improvements in global health. It was one of the interventions highlighted by the Lancet Commission on Investing in Health to bring about ‘health convergence’—whereby countries with the poorest mortality rates are brought up to the level of the highest performing middle-income countries.^11^ Gains for recipient countries through vaccination may be amplified by knock-on effects: return on investment estimates for projected immunisation levels in developing countries over the current decade are up to 16 times the initial costs.^12^

Our research answers the following key questions: did Gavi support bring an increase in countries’ immunisation rates beyond what would have occurred in its absence? What size of impact has Gavi had? Did Gavi also have a knock-on impact on child mortality rates? Given that the institution’s core work is to improve access to vaccines, we might expect to find vaccination rates rising as a matter of course. However, this is by no means certain. Had Gavi not been created, countries could have found other ways to provide vaccine access for their populations. Alternatively, Gavi funding could have crowded out domestic spending. Some studies have found that DAH is highly fungible, although other evidence is not so clear-cut.^13–15^ Vaccines are of course a means to an end: better health and reduced mortality through lowered disease incidence. Any positive changes in vaccination levels would be expected to improve infant and child mortality.^16^ In addition, protective efficacy may be compounded by non-specific immunisation effects.^17 18^ These expectations need to be empirically checked. As well as the effectiveness of health aid,^19 20^ this paper also speaks to the literature on the link between interventions and population health.^21–25^

Aside from Gavi’s own evaluations, two existing independent studies have looked at the effects of Gavi support on immunisation coverage. Early research showed positive impacts from Gavi funding on diphtheria, pertussis and tetanus (DPT) immunisation rates.^26 27^ The data and methods used have become dated, though, and the analysis only looked at this one vaccine. More recently, another study found that Gavi had null or insignificant impacts on coverage of four out of five vaccines.^28^ Compared with these previous studies, our work extends both the time frame over which impacts were estimated—allowing for longer term effects—and the methods used. Although we interpret our main results as causal, our use of non-experimental data means we cannot exclude with absolute certainty the possibility that some bias remains in our estimates. However, we perform an extensive list of checks to maximise our confidence in the results.

## Methods

### Data sources

We use a country-level panel dataset covering 1995–2016. Our core variables are taken from the World Bank World Development Indicators (WDI, February 2018 version). The WDI provide annual information on several country characteristics such as population size, purchasing power parity (PPP)-adjusted gross domestic product (GDP) per capita, primary school enrolment rates and infant and under-five mortality rates. They also provide WHO/UNICEF figures on DTP3 (third dose of a DPT-containing vaccine, such as tetravalent or pentavalent) and MCV1 (first dose of a measles-containing vaccine) immunisation rates for surviving children aged 12–23 months old, which are our main outcome variables. Gavi supports other vaccines, but we focus on these two measures for which cross-country longitudinal data is most reliably and widely available, including prior to Gavi’s creation. DPT and measles are also two of the earlier vaccines that Gavi supported. Since the data measure vaccinations administered to children aged under 12 months, that is, the previous year, we shift the rates back by 1 year, to match up to when the vaccinations actually took place. Although late ‘catch-up’ vaccinations do take place, these are most often late by just a matter of weeks and hence it is reasonable to consider immunisation rates at the appropriate age.^29^

The World Bank’s historical country classifications enable us to identify low-income and lower-middle-income countries in 2000 for our analysis sample. We have 5 years of data preceding the creation of Gavi and 16 years following. The list of countries eligible for Gavi support and its evolution over the years is collated from the Gavi website and public reports. We exclude countries that lost eligibility during our study period from the main analysis. We also exclude East Timor, Kosovo and South Sudan, which became independent nations during our analysis time period. There are 51 countries in our always-eligible treatment group and 37 countries in the never-eligible control group (online supplementary tables 1–2).

10.1136/bmjgh-2019-001789.supp1 Supplementary data

### Patient and public involvement

Patients were not involved.

### Statistical analysis

Gavi supports vaccination programmes in countries below a per capita gross national income (GNI) threshold, which from 2000 to 2011 was US$1000. Over time, Gavi has updated its policy, with the threshold for 2018 eligibility (GNI per capita 2014–2016 average, World Bank Atlas method) set at US$1580. An eligibility cut-off lends itself to the regression discontinuity design (RDD) used by Dykstra and colleagues.^28^ However, this method constrains the analysis to estimate effects only on countries near the threshold—ignoring Gavi’s impact on poorer countries. In the resulting relatively small country panel dataset, an RDD approach lacks statistical power to detect significant impacts.^30^ Expanding the sample bandwidth too far increases power but compromises internal validity.^31^

Instead, we follow a quasi-experimental difference-in-differences identification strategy comparing the evolution of immunisation rates in Gavi-eligible countries to similar countries not eligible for support.^32–34^ The focus on changes over time allows us to remove the influence of country fixed characteristics correlated with eligibility and vaccination outcomes. Not all eligible countries receive Gavi immunisation assistance in every year (ie, there are no Gavi disbursements for some eligible countries in some years, although this is rare), since countries must apply for funding and applications are subject to review.^35^ Our analysis, therefore, considers eligibility for Gavi funding as the ‘treatment’, similar to an intent-to-treat model. We follow this approach because whether countries did get funding—and how much—is endogenous to country characteristics such as institutional capacity and health burdens. An alternative would be an attributed vaccine impact model for mortality outcomes. However, BenYishay and Kranker detail how these epidemiological models suffer from a number of limitations.^36^

#### Main specification

To quantify the Gavi impact, we use the following specification with country and year fixed effects for outcome variable *y*:

(1) $$y_{ct}=\alpha_{c}+\delta_{t}+\beta_{1}D_{ct}+X_{ct}^{\prime }\beta_{2}+e_{ct}$$

where *c* indexes countries and *t* years. $D_{ct}$ is a binary variable equal to 1 if country *c* was eligible to receive Gavi support in year *t* and zero otherwise. $\alpha_{c}$ and $\delta_{t}$ are country and year fixed effects, respectively. $X_{ct}$ is a vector of country characteristics that explain variation in immunisation rates. It includes PPP-adjusted GDP per capita (which is strongly correlated with, and a good proxy for, other human development outcomes) and population in our baseline model. Our identification strategy requires the error, $e_{ct}$, to be uncorrelated with the treatment, $D_{ct},$ conditional on all the control variables. This might not hold if there are other time-varying differences between countries that are correlated with Gavi eligibility and the health outcomes. In some alternative specifications, we add additional control variables to check the sensitivity of our results. However, this reduces our sample size and could skew our results if systematically certain types of countries have missing data. Also, since most country-level control variables are measured with some error, including those affects the consistency of the estimates. Despite this, the extended specification is our preferred model for the infant and child mortality outcomes, since the number of potential confounders is very likely higher. It is indeed possible that treatment countries implemented health reforms and benefitted from other public interventions during our study period. We account for this possibility in the robustness tests section where we control for various other sources of international assistance and health aid. The main coefficient of interest is $\beta_{1}$ measuring the effect the introduction of Gavi has had on each outcome. We estimate Equation 1 by ordinary least squares using the Statistical analysis software *Stata* (V.15.1). SEs are clustered by country.^37^

In 2000, Gavi offered assistance to all low-income countries and some lower-middle-income countries. For a meaningful comparison we restrict our counterfactual control group to only lower-middle-income non-eligible countries. The key identifying assumption is that in the absence of Gavi, vaccination rates in eligible and control countries would have evolved in a similar way (parallel trends). We have sufficient data prior to 2000 to test whether this assumption is plausible.

The comparison group we use is not perfect and there might be differences between the treatment and control countries. However, the difference-in-differences research design cancels the influence of time fixed characteristics that may differ between the two groups. We address potential concerns stemming from differences in immunisation and income levels between the groups by checking results when the sample is restricted to countries closer in baseline levels. To allay other concerns, we run a suite of sensitivity and robustness checks.

#### Parallel trends test

Since the internal validity of our analysis relies on the parallel trend assumption, we provide a formal Granger type of causality test.^32^ The intuition is that consequences should happen only after their causes, not vice versa. Therefore, the difference between the trends in outcome variables for the two groups of countries (treatment and control) prior to 2000 should not be statistically different from zero, that is, the trends follow parallel paths. For our study, the impact of Gavi on immunisation rates should only be observed after its creation, with zero effect in the late 1990s from future eligibility to Gavi support. To implement this test, we estimate the following model:

(2) $$y_{ct}=\alpha_{c}+\delta_{t}+\sum_{\tau =2}^{5}\lambda_{+\tau }D_{c,t+\tau }+\sum_{\tau =0}^{10}\lambda_{-\tau }D_{c,t-\tau }+X_{ct}^{\prime }\beta_{2}+e_{ct}$$

where the sums allow, respectively, for anticipatory (leads) and post-treatment (lags) effects. We leave out the last pretreatment year (1999), and all the leads and lags are expressed relative to this omitted year serving as the baseline.

**Figure 1:**
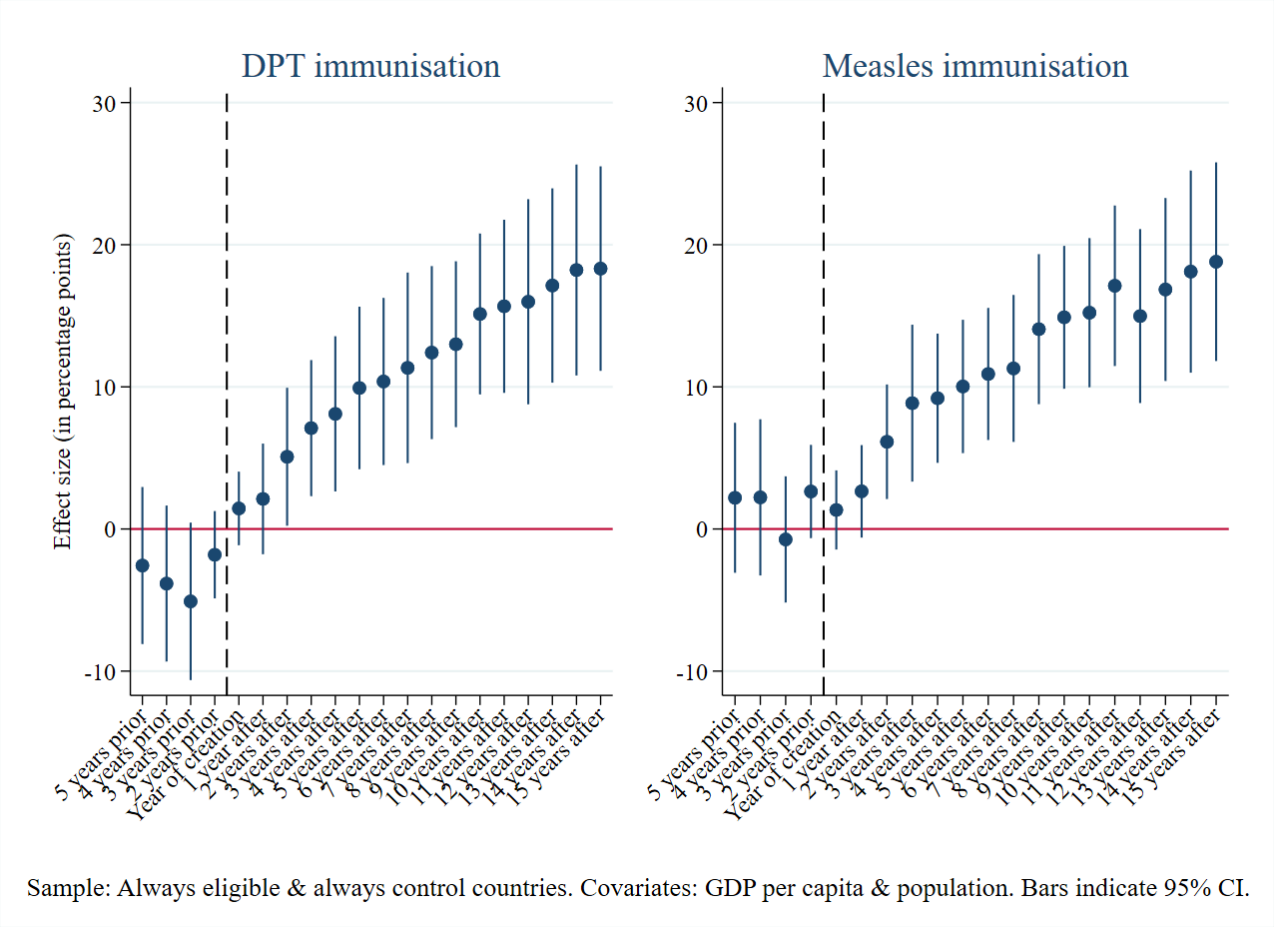
Diptheria, pertussis and tetanus (DPT) and measles immunisation rates: parallel trends Granger causality leads and lags test.

**Figure 2:**
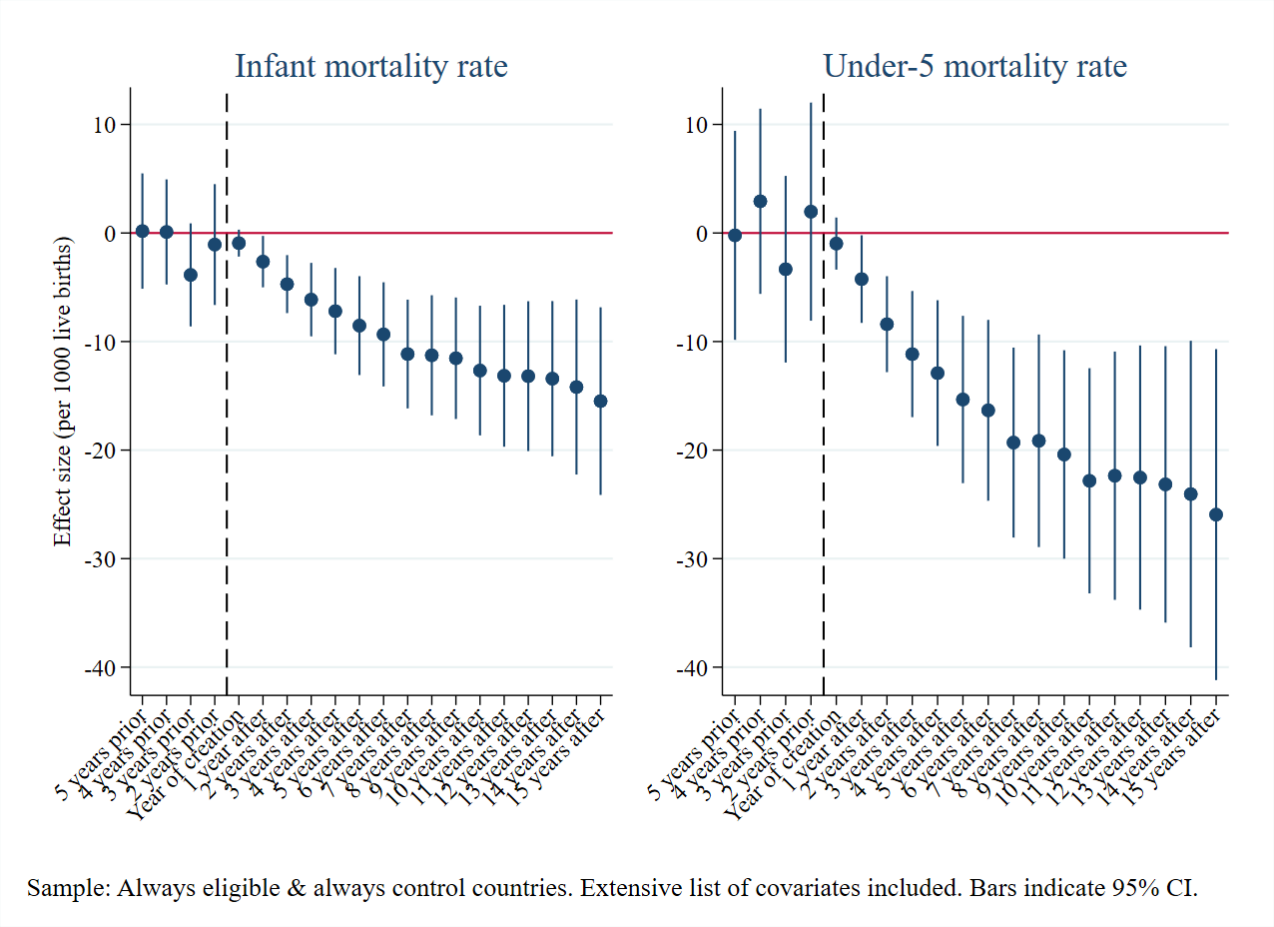
Under-five and infant mortality rates: parallel trends Granger causality leads and lags test.

For both DPT and measles immunisations, our estimates show no significant effect on the rates in the years preceding the establishment of Gavi but increasingly significant and positive effects thereafter (figure 1). These patterns are consistent with a causal interpretation, and confirm the validity of the difference-in-differences model using this control group of countries. We run the same test for our two mortality outcomes (figure 2), with similarly strong results. These results also support our choice of covariates to include in the preferred models in each case.

## Results

Overall, we find solid evidence that Gavi has helped improve protection against DPT and measles in developing countries (table 1). Our point estimates show that on average Gavi increased the DPT immunisation rate by 12.02 percentage points (95% CI 6.56 to 17.49) over the study period. Rates of vaccination against measles were boosted by 8.81 percentage points (95% CI 3.58 to 14.04). These average treatment effects are on top of baseline 1995–1999 mean immunisation rates of 70.95% and 71.65% for DPT and measles respectively, equating to average 16.94% (DPT) and 12.30% (measles) increases on baseline levels. With some countries eligible for Gavi funding not receiving any in some years, the true difference between treated and control countries is probably even greater than the observed difference, making these values lower bounds on the true effects. It should be noted that we estimate the effect of Gavi coming into existence; these are not impacts of scaling up Gavi’s operations. It is true that the estimated effects could include impacts from other policies and programmes starting at the same time, if these were also directed just to Gavi-eligible countries. However, we go on to run a suite of checks to eliminate this possibility as much as possible.

**Table 1:** Gavi effect on immunisation outcomes

	Full sample, unweighted	Full sample, population weighted†	Full sample, controlling for GNI growth	Full sample, controlling for domestic health public spending‡	Full sample, controlling for wide set of additional covariates§	Subsample with richer Gavi-eligible and poorer Gavi ineligible countries¶	Subsample with higher average baseline immunisation rate††	Subsample with baseline immunisation rate below 90%‡‡	Extended sample, including late graduate countries
Panel A: Dependent variable: immunisation rate, DPT (% 12–23 months old)									
Gavi eligibility effect	12.02***	11.03***	12.99***	11.91***	6.82**	13.88***	7.21***	11.47***	9.18***
	(2.75)	(2.62)	(2.80)	(2.73)	(2.94)	(4.03)	(2.40)	(3.18)	(2.61)
Observations	1735	1714	1211	1712	1096	650	1224	1363	2134
Number of countries	84	83	69	84	82	31	59	66	103
Adjusted R-squared	0.36	0.59	0.47	0.38	0.36	0.30	0.17	0.43	0.33
Panel B: Dependent variable: immunisation rate, measles (% 12–23 months old)									
Gavi eligibility effect	8.81***	11.11***	9.44***	8.15***	4.96*	9.76**	7.31***	8.81**	8.09***
	(2.63)	(2.62)	(2.54)	(2.55)	(2.76)	(4.01)	(2.42)	(3.34)	(2.38)
Observations	1735	1714	1211	1712	1096	650	1493	1342	2134
Number of countries	84	83	69	84	82	31	72	65	103
Adjusted R-squared	0.30	0.62	0.44	0.32	0.34	0.19	0.20	0.36	0.28
Country fixed effects	Yes	Yes	Yes	Yes	Yes	Yes	Yes	Yes	Yes
Year fixed effects	Yes	Yes	Yes	Yes	Yes	Yes	Yes	Yes	Yes
Covariates	Yes	Yes†	Plus GNI growth	Plus domestic spending	Wider set	Yes	Yes	Yes	Yes

Robust SEs in parentheses clustered at the country level.

Main data source: World Development Indicators. Sample period: 1995–2016. All regressions include GDP per capita (in log) and population (in log) unless otherwise stated.

***p<0.01, **p<0.05, *p<0.1.

†Column 2: Covariates only include PPP-adjusted GDP per capita (in log), weights are equal to birth cohort size in 2000.

‡Column 4: Domestic general government health expenditure as % GDP (WHO Global Health Expenditure Database). No observations available on domestic expenditure prior to 2000. Public expenditure data for 1995–1999 set to 2000 value.

§Column 5: Additional covariates are: primary school enrolment, secondary school enrolment, urban population (World Development Indicators) and political rights (Freedom House political rights index, available in the QoG standard dataset: January 2017 version).

¶Column 6: Eligible (non-eligible) countries in the bottom (upper) half of the GNI per capita distribution in 2000 excluded.

††Column 7: Countries with average baseline DPT (measles) immunisation rates below 60% (50%) excluded.

‡‡Column 8: Countries with baseline immunisation rate above 90% excluded.

DPT, diphtheria, pertussis and tetanus; GDP, gross domestic product; GNI, gross national income; PPP, purchasing power parity.

In this model, each country carries the same weight regardless of its size. Larger, more populous countries usually receive more funding from Gavi. Population-weighted results by birth cohort size are shown in column 2. The point estimates are similar and indicate a strong and significant impact on coverage of both immunisations. This also holds when using total population size as weights. Our positive impacts are robust to controlling for a broad range of additional covariates, including the rate of economic growth (column 3), domestic health public spending (column 4) and—although with a reduction in point estimate—primary and secondary education enrolment, urbanisation and quality of institutions (column 5).

Gavi began supporting MCV2 (second dose) measles vaccinations specifically in 2004 and MCV1 in 2012. The earlier Gavi-supported second dose vaccine is used as an opportunity to administer a first dose for children who are not already vaccinated.^38 39^ However, Gavi’s funding of general immunisation system support and supply chain strengthening took effect from the start, and Gavi channelled non-country-specific funding for measles vaccination programmes via several partner organisations. As an additional check, we estimate the Gavi effect on measles vaccination coverage from 2004. These results show a slightly larger Gavi impact of 10.54 percentage points (95% CI 6.09 to 15.00).

In our sample of low-income and lower-middle-income countries, there is considerable variation in socioeconomic development and hence GNI per capita. The more advanced control countries might have differed in unobserved characteristics from the less advanced treatment countries (hence not comparing like with like). Alternatively, the lowest income countries could simply be ‘catching up’—convergence—rather than experiencing a Gavi effect. Therefore, we conduct the first in a series of heterogeneity analyses, replicating our estimation while excluding countries above the median GNI per capita of never-eligible countries in 2000 and countries below the median of eligible countries. Working with this reduced sample does not affect our conclusions (column 6). In fact, we found larger point estimates for Gavi’s impacts, although also wider CIs.

Another concern is that our results might be driven by eligible countries with very low baseline immunisation rates for which improving access to vaccines would be easier. To address this, we exclude all countries with average DPT vaccination rates for 1995‒1999 below 60% and similarly for measles vaccination rates below 50%. All the never-eligible control countries had vaccination rates above those cut-off levels prior to 2000. Although the estimated effect size decreases, our results still indicate that Gavi support has contributed to increasing levels of DPT and measles vaccination (column 7). Our results remain robust to adjusting the cut-off level. It should be noted that there is a wide spread of baseline coverage rates in both our control and treatment groups. It is not simply the case that countries ineligible for Gavi assistance were already at a maximum possible ‘ceiling’ for vaccination rates. Testing this more formally, we estimate the Gavi treatment effect for a subsample of countries excluding those with baseline vaccination rates above 90%. These results are very close to the main estimates (column 8). This also removes concerns about using a linear identification method for bounded outcomes.

The 20 late graduate countries, which all became ineligible for funding during Gavi’s third phase (2011–2015), are excluded from our main analysis. However, these countries did continue to receive Gavi support for existing programmes after graduation and arguably they form part of the impact of Gavi funding. The point estimates when including these countries (column 9) are only slightly smaller than the main results and actually underestimate the true impact, given that some late graduate countries were not funded by Gavi through to 2016.

The other Gavi-supported immunisation for which there is data pre-2000 is hepatitis B. However, these data contain large gaps with many missing observations. Despite the limitations, we still assess Gavi’s impact—since hepatitis B is one of the diseases in which Gavi has invested the most. We find a positive average effect on vaccination rates, but the point estimate is high, at 25.12 percentage points, with large SEs (95% CI 0.47 to 49.77). While a positive impact on hepatitis B immunisation coverage is consistent with our previous findings, the estimation is imprecise. Another vaccine that Gavi supported early on is against Haemophilus influenzae type B (Hib), but data coverage is seriously limited. There are only 2.27% of country–year observations available prior to 2000, which prevents us from analysing Gavi’s impact on Hib vaccine rates.

Despite endogeneity concerns from Gavi funding decisions, for comparison we also run a variable treatment model to measure the Gavi effect depending on level of funding disbursed per year (measured in real 2016 US$ million).^32^ For both immunisations, we estimate that increasing Gavi disbursements by US$100 million led to a significant rise in vaccination rates over our study period: an average increase of 15.45 percentage points (95% CI 4.74 to 26.15) for DPT and 11.99 percentage points (95% CI 0.76 to 23.22) for measles.

While Gavi’s objective is to provide access to new and underused vaccines, its long-term mission is to save children’s lives. Despite our positive immunisation coverage results, there could have been no impact on mortality if, for instance, Gavi had prioritised vaccines not relevant to address specific health burden needs or had crowded out other life-saving health expenditures. Table 2 provides our estimates of the effect from Gavi support on infant and under-five mortality rates, using the same regression model as for the immunisation results. Given the greater potential confounding factors for mortality than for immunisation coverage, the impact coefficients when controlling for a wider set of covariates (column 5) are the more appropriate baseline estimates of the true Gavi effect. Taking this approach, we can say that Gavi eligibility led to an average reduction of 6.22 (95% CI −10.47 to −1.97) infant and 12.23 (95% CI −19.66 to −4.79) under-five child deaths per 1000 live births over our study period. With pretreatment 1995–1999 means of 66.45 infant and 101.42 child deaths per 1000 in our sample, this impact corresponds to an average 9.36% reduction in the infant mortality rate and an average 12.06% reduction in the under-five mortality rate. These reductions are not solely due to DTP and measles vaccines, but Gavi’s support of and introduction of many new vaccines, plus improvements in immunisation and health systems. The mortality results hold strongly if we control for the rate of economic growth (as shown in column 3). This is important because domestic growth may lead to improved health outcomes.^20^

**Table 2:** Gavi effect on mortality outcomes

	Full sample, unweighted	Full sample, population weighted†	Full sample, controlling for GNI growth and wide set of additional covariates§	Full sample, controlling for domestic health public spending and wide set of additional covariates§‡	Full sample, controlling for wide set of additional covariates§	Subsample with richer Gavi-eligible and poorer Gavi ineligible countries§¶	Subsample with higher average baseline measles immunisation rate§††	Subsample with baseline measles immunisation rate below 90%§‡‡	Extended sample, including late graduate countries§
Panel A: Dependent variable: infant mortality rates (per 1000 live births)									
Gavi eligibility effect	−11.92***	−15.02***	−7.08***	−6.29***	−6.22***	−1.05	−7.07***	−9.33***	−6.69***
	(2.32)	(2.30)	(1.86)	(2.22)	(2.14)	(2.38)	(2.50)	(2.18)	(1.69)
Observations	1823	1801	812	1085	1096	403	926	807	1313
Number of countries	84	83	67	82	82	30	70	63	101
Adjusted R-squared	0.79	0.88	0.82	0.79	0.79	0.80	0.77	0.79	0.80
Panel B: Dependent variable: under-five mortality rates (per 1000 live births)									
Gavi eligibility effect	−22.46***	−29.99***	−14.97***	−12.25***	−12.23***	−5.22	−15.14***	−17.24***	−13.18***
	(4.39)	(4.52)	(3.46)	(3.91)	(3.74)	(4.30)	(4.34)	(3.92)	(3.08)
Observations	1823	1801	812	1085	1096	403	926	807	1313
Number of countries	84	83	67	82	82	30	70	63	101
Adjusted R-squared	0.76	0.82	0.81	0.79	0.78	0.74	0.75	0.80	0.78
Country fixed effects	Yes	Yes	Yes	Yes	Yes	Yes	Yes	Yes	Yes
Year fixed effects	Yes	Yes	Yes	Yes	Yes	Yes	Yes	Yes	Yes
Covariates	Yes	Yes†	Wider set plus GNI growth	Wider set plus domestic spending	Wider set	Wider set	Wider set	Wider set	Wider set

Robust SEs in parentheses clustered at the country level.

Main data source: World Development Indicators. Sample period: 1995–2016. All regressions include GDP per capita (in log) and population (in log) unless otherwise stated.

***p<0.01, **p<0.05, *p<0.1.

†Column 2: Covariates only include PPP-adjusted GDP per capita (in log), weights are equal to birth cohort size in 2000.

‡Column 4: Domestic general government health expenditure as % GDP (WHO Global Health Expenditure Database). No observations available on domestic expenditure prior to 2000. Public expenditure data for 1995–1999 set to 2000 value.

§Columns 3 to 9: Additional covariates are: primary school enrolment, secondary school enrolment, urban population (World Development Indicators) and political rights (Freedom House political rights index, available in the QoG standard dataset: January 2017 version).

¶Column 6: Eligible (non-eligible) countries in the bottom (upper) half of the GNI per capita distribution in 2000 excluded.

††Column 7: Countries with average baseline measles immunisation rates below 60% excluded.

‡‡Column 8: Countries with baseline immunisation rate above 90% excluded.

GDP, gross domestic product; GNI, gross national income; PPP, purchasing power parity.

There are of course other sources of health aid, and it is possible that at the same time as Gavi was created, there was a separate movement encouraging vaccination programmes. Estimating Gavi’s average impact on immunisation rates while controlling for all other multilateral and Development Assistance Committee bilateral DAH gives very similar results to the main point estimates—11.94 percentage points (CI 6.48 to 17.39) for DPT immunisation coverage and 8.75 percentage points (95% CI 3.50 to 13.99) for measles. Two years after the creation of Gavi, the Global Fund to Fight AIDS, Tuberculosis and Malaria was established, also working with developing countries. We also estimate the Gavi effect just controlling for Global Fund annual disbursements and find almost identical results. We conclude that our estimates of Gavi’s vaccinations impact are not driven by other aid activities. This also holds for our infant and child mortality results. (See online supplementary table 3.)

Since 1977, Latin American countries have benefitted from the Pan American Health Organisation (PAHO) and its Revolving Fund for essential vaccines, syringes and other supplies. To make sure that we have not overestimated Gavi’s impacts by absorbing a PAHO effect, we exclude all Latin American countries, finding even larger positive, significant Gavi effects. This is not surprising given that more PAHO-assisted countries are in our control sample than in our treatment group. Another potentially competing vaccination health programme we need to consider is the Measles Initiative (later the Measles & Rubella Initiative), set up in 2001. However, Gavi actually helps fund the Measles & Rubella Initiative, having channelled US$197 million through the organisation between 2005 and 2008 alone.^40^ Although there are other funders, Gavi is one of the two largest contributors, and we expect its support for the initiative to have contributed to the measles impacts we find.^40^

At around the same time as Gavi was created, the Heavily Indebted Poor Countries (HIPC) Initiative approved debt relief for a number of Gavi-eligible countries. It is therefore possible that our estimates of Gavi’s impact could be picking up a HIPC effect on health outcomes in recipient countries. We rerun our baseline analysis adding in two alternate definitions of HIPC support. Our immunisation results are highly robust to these specifications (online supplementary table 4).

To strengthen the credibility of our results, we run a falsification test. We assess Gavi’s impact on incidence or prevalence of diseases it has not targeted: HIV/AIDS, malaria and tuberculosis (while controlling for Global Fund disbursements, since these are all diseases that organisation does target). Confirming our expectations, we fail to find any significant effect of Gavi disbursements on these disease measures (online supplementary table 5).

In addition to inevitable measurement error for aggregated statistics, Gavi-recipient countries have been accused of inflating DPT vaccine administrative data used to construct coverage indicators because they receive extra funding under the flexible immunisation services support programme based on the number of children receiving vaccination. The cash reward is thought to have encouraged governments and health service providers to over-report immunisation coverage.^41 42^ To check our results, we replicate the analysis using immunisation rates from Demographic and Health Surveys (DHS), which data are gathered via individual and household surveys (online supplementary table 6). Coverage of DHS surveys is more limited, though, which makes precision difficult. We estimate that in Gavi-eligible countries the percentage of children with no vaccination at all fell by 5.13 percentage points (CI −8.46 to −1.79), DPT vaccination rates increased by 8.23 percentage points (2.46 to 14.01) and measles vaccine coverage increased by 11.16 percentage points (95% CI 5.15 to 17.17). These additional DHS results provide evidence that our main results are not due to misreporting.

## Discussion

In this study, we find a large and positive total average effect of Gavi support on access to measles and DPT vaccinations, across all Gavi-eligible countries. Our statistical method covers the full range of aid-recipient countries, unlike the most recent Gavi study by Dykstra and colleagues, allowing for better estimation.^28^ While we cannot entirely rule out the possibility that our results may be biased due to unobservable time-varying characteristics and interventions, we run an extensive list of robustness checks giving us strong confidence in the internal validity of our findings. Our results are also sufficiently powered. We provide results for two immunisations—those for which data are most widely available—and a third supportive result (Hepatitis B). DPT and measles are early vaccines that Gavi supported, though, and it is possible that Gavi’s impacts for newer, more expensive immunisations may differ.

Our positive, significant DPT and measles results are in contrast to the insignificant vaccine results found by Dykstra and colleagues.^28^ We ascribe this to a lack of statistical power in their RDD analysis rather than the fact that they can only consider richer Gavi-eligible countries, since we do not find differential effects estimating separately for countries in the upper versus lower GDP per capita income distribution. This somewhat contradicts the suggestion made by Dykstra and colleagues that richer Gavi-eligible countries do not see large impacts on DPT immunisation rates, but that poorer countries may. Aside from the aforementioned statistical power issues, this could also be because we look at outcomes over a long-run time frame. Repeating our baseline difference-in-difference analysis but using Dykstra and colleagues’ method to define the subsamples of treatment and control countries, we find statistically significant positive effects on DPT and measles immunisation coverage, with point estimates around half the size of our baseline estimates (online supplementary table 7). Our own subsample analyses show that there are more gains in vaccination coverage to be made from Gavi support for countries with lower baseline immunisation rate rather than simply poorer countries. The converse is true for impacts on mortality.

To investigate whether Gavi’s impact faded or increased over time for these early vaccinations, we collapse our dataset into four time periods and estimate separate treatment effects. The periods correspond to the pretreatment years 1995–1999 followed by the three Gavi phases: I (2000–2006), II (2007–2010) and III (2011–2015). The positive impact on immunisations rates has grown over time, consistent with increasing disbursements from Gavi (online supplementary table 8). Despite this, rough calculations suggest that this increase is due to higher levels of funding in later phases rather than an increase in the impact per dollar spent. In fact, our calculations are suggestive of a decrease in impact per dollar over time, particularly for DPT, as Gavi’s impact was found to increase at a slower rate than disbursements. This is likely due to Gavi’s focus on the newer but more expensive tetravalent and pentavalent vaccines, which include DPT. When we split Gavi funding into systems support and vaccine support and run a continuous effects model, we find that general funding in fact has a larger impact on vaccination coverage than new and underused vaccine support (NVS) (online supplementary table 9). This is in good part due to the fact that Gavi disburses much more for NVS.

Saving lives is the longer-term aim from the creation of Gavi. Calculations based on our point estimate of a reduction in infant mortality of 6.22 per 1000 live births over the period to 2016, produce an estimate of lives saved very similar to Gavi’s own figure of 9 million. By multiplying Gavi’s impact on under-five mortality per US$ disbursed over the study period by the number of such deaths we roughly calculate the cost for Gavi to save one child’s life at $118. Our most conservative estimate for the statistical value of life is more than 500 times the cost for Gavi to save an under-five. Mortality improvements will not be solely due to prevention of deaths from diseases for which Gavi supports vaccinations—even though the fund expanded coverage to 13 different diseases over the sample time period. The Gavi effects on infant and under-five mortality may be compounded by non-specific effects from measles immunisation.^17 18^ In addition, Gavi’s support of in-country immunisation systems as well as potential knock-on effects of improved health on recovery from other diseases help bring down mortality rates.^43–45^ Also, other public health programmes can be attached to vaccination campaigns.^46^ In the case of some fungibility of Gavi-provided assistance, recipient countries might have shifted domestic resources to other health programmes that also reduce mortality. If the net result is still population health improvement, though, some DAH fungibility may be seen as acceptable.

### Transition

In addition to Gavi’s assistance, Gavi-supported countries must contribute a set amount per vaccine dose. In this way, national ownership is built while Gavi bears the majority of the costs. As an economy grows above the World Bank’s low income threshold, it enters a preparatory transition phase, and the national vaccine contribution starts to rise.^47^ Once it has grown beyond Gavi’s income eligibility threshold, that country enters a 5-year accelerated transition process to phase out Gavi support. During this period, Gavi looks to ensure sustainability of immunisation programmes.^48^ Existing vaccine programmes are supported but new applications are only accepted for strengthening routine immunisations or equity.^35^ In 2016, 16 countries were in transition and by 2020 a further 12 countries are expected to have transitioned.^10^ While our analysis estimates the effect of the introduction of Gavi, it also provides a cautionary note on the management of transition processes. The size of Gavi’s impacts on immunisation coverage and mortality rates are indicative of the size of the problem that transitioning middle-income countries may face. Removal of support is not simply the opposite of its introduction, but there is no other estimate available at present. Not enough countries have gone through transition with sufficient post-transition time periods.

Reviewing individual country vaccination rates across transition, most countries’ DPT immunisation coverage either remained relatively stable or dropped off slowly (online supplementary figure 1). Comparing effects estimated from alternative treatment groups of countries including/excluding late graduating countries and different time periods including/excluding transition, preliminary calculations suggest that, on average and in the short run, countries losing Gavi support under the current managed transition policy do see a relative drop in immunisation rates (online supplementary table 10). However, this is not as large a (negative) impact as the (positive) impact of Gavi’s introduction.

Gavi’s vaccine-purchasing aid is found to be fungible in analysis based on countries close to the transition GNI per capita cut-off.^28^ Unfortunately, data showing domestically sourced government spending on vaccines is not available for many countries, and we cannot adjust for this in our analysis. However, Dykstra and colleagues’ results do complement our suggestive evidence for lesser transition impacts and reduced impacts per dollar over time, which could be showing less-poor countries’ ability to domestically absorb some Gavi funding slack. Further research on the transition issue is warranted.

## Conclusion

In this case, the verticalisation of aid through the creation of a specialised, immunisation-focused global fund has had positive outcomes. In part, this may be due to Gavi’s work in vaccine provision—research and development support, price support and supply chain support—as well as the direct impacts on immunisation coverage. Vaccines are global public goods, for which supranational coordination is invaluable.^10 11^ Other potential sectors for verticalisation may not have this property and Gavi’s success does not necessarily provide a template. The question of transition out of receiving health aid is also a significant one that should be looked at in more depth as more data become available.
