# Supplementary material for: Has Gavi lived up to its promise? Quasi-experimental evidence on country immunisation rates and child mortality
Source: BMJ Glob Health. 2019 Dec 3;4(6):e001789. doi: 10.1136/bmjgh-2019-001789 (PMC6936423; doi:10.1136/bmjgh-2019-001789)
Supplement: Supplementary data [file bmjgh-2019-001789supp001.pdf]

Online Supplementary Appendix

Table 1: Low and lower-middle income country groupings according to Gavi eligibility status

Never eligible (control)		Always eligible (treatment)		Early graduates (not in main sample)	Late graduates (not in main sample)
Algeria	Micronesia, Fed. Sts.	Afghanistan	Lesotho	Albania	Angola
Belarus	Morocco	Bangladesh	Liberia	Bosnia & Herzegovina	Armenia
Belize	Namibia	Benin	Madagascar	China	Azerbaijan
Cabo Verde	Paraguay	Burkina Faso	Malawi	Turkmenistan	Bhutan
Colombia	Peru	Burundi	Mali		Bolivia
Dominican Republic	Philippines	Cambodia	Mauritania		Congo, Rep.
Ecuador	Russian Federation	Cameroon	Mozambique		Cuba
Egypt, Arab Rep.	Samoa	Central African Republic	Myanmar		Georgia
El Salvador	St. Vincent & the Grenadines	Chad	Nepal		Guyana
Equatorial Guinea	Suriname	Comoros	Niger		Honduras
Fiji	Swaziland	Congo, Dem. Rep.	Nigeria		Indonesia
Guatemala	Syrian Arab Republic	Cote d'Ivoire	Pakistan		Kiribati
Iran, Islamic Rep.	Thailand	Djibouti	Rwanda		Moldova
Iraq	Tonga	Eritrea	Sao Tome & Principe		Mongolia
Jamaica	Tunisia	Ethiopia	Senegal		Nicaragua
Jordan	Vanuatu	Gambia, The	Sierra Leone		Papua New Guinea
Kazakhstan	West Bank & Gaza	Ghana	Solomon Islands		Sri Lanka
Macedonia, FYR		Guinea	Somalia		Ukraine
Maldives		Guinea-Bissau	Sudan		Uzbekistan
Marshall Islands		Haiti	Tajikistan		Vietnam
		India	Tanzania		
		Kenya	Togo		
		Korea, Dem. People's Rep.	Uganda		
		Kyrgyz Republic	Yemen, Rep.		
		Lao PDR	Zambia		
			Zimbabwe		

Table 2: Summary statistics

Variable	Country-year observations (n)	Mean	SD	Country-year observations (n)	Mean	SD
	Gavi eligible countries			Control group of non-eligible countries		
Immunization, DPT (% 12–23 months old)	1,114	71.56	19.92	792	87.10	13.96
Immunization, measles (% 12–23 months old)	1,114	70.55	18.30	792	87.59	12.85
Infant mortality rate (per 1,000 live births)	1,122	70.00	25.49	814	26.61	16.12
Under-5 mortality rate (per 1,000 live births)	1,122	107.42	47.31	814	33.49	24.13
Population (million)	1,117	45.92	161.07	814	19.82	31.44
GDP per capita, constant PPP US$	1,059	763.60	397.07	786	4332.98	2435.38
Gavi disbursements, constant US$ million	1,122	7.77	18.51	814	0	0
Global Fund disbursements, constant US$ million	1,122	19.88	39.40	792	3.26	7.16
Global DAH disbursements (net of Gavi), constant US$ million	1,122	69.71	105.75	792	15.99	34.57
HIV prevalence, total (% of population ages 15–49)	1,056	3.49	4.96	572	2.25	5.76
Tuberculosis (TB) incidence, per 100,000 people	867	266.69	195.80	629	121.22	206.60
Incidence of malaria (per 1,000 population at risk)	200	218.53	184.48	76	35.23	76.00

Data source: WDI, OECD CRS, Gavi.

Jaupart P, *et al. BMJ Global Health* 2019; 4:e001789. doi: 10.1136/bmjgh-2019-001789

Table 3: Gavi effect, controlling for other DAH

	(1) Controlling for Global Fund disbursements	(2) Controlling for all non-Gavi DAH	(3) Excluding Latin American (PAHO) countries	(4) Controlling for Global Fund disbursements	(5) Controlling for all non-Gavi DAH	(3) Excluding Latin American (PAHO) countries
	Panel A: Dependent variable – Immunization rates					
	DPT (% 12–23 months old)			Measles (% 12–23 months old)		
Gavi eligibility effect	11.29***	11.94***	13.86***	7.93***	8.75***	10.94***
	(2.60)	(2.74)	(3.25)	(2.42)	(2.64)	(3.16)
Observations	1,714	1,714	1,484	1,714	1,714	1,484
Number of countries	83	83	72	83	83	72
Adjusted R-squared	0.37	0.37	0.37	0.32	0.31	0.33
	Panel B: Dependent variable – Mortality rates					
	Infant mortality (per 1,000 live births)			Under-5 mortality (per 1,000 live births)		
Gavi eligibility effect	-5.62**	-6.20***	-5.92**	-11.29***	-12.23***	-10.26**
	(2.17)	(2.13)	(2.33)	(3.75)	(3.70)	(4.05)
Observations	1,080	1,080	901	1,080	1,080	901
Number of countries	81	81	70	81	81	70
Adjusted R-squared	0.80	0.79	0.80	0.80	0.79	0.80
Country fixed-effects	Yes	Yes	Yes	Yes	Yes	Yes
Year fixed-effects	Yes	Yes	Yes	Yes	Yes	Yes
Covariates	Yes	Yes	Yes	Yes	Yes	Yes

Robust standard errors in parentheses clustered at the country level. *** p<0.01, ** p<0.05, * p<0.1. Main data source: World Development Indicators. Sample period: 1995–2016. All regressions include PPP-adjusted GDP per capita (in log) and population (in log) unless otherwise stated. Panel B: Additional covariates are: primary school enrolment, secondary school enrolment, urban population, and political rights.

Table 4: HIPC debt relief initiative

Dependent variables:	(1) Immunization, DPT (% 12-23 months old)	(2) Immunization, measles (% 12-23 months old)	(3) Infant mortality rate (per 1,000 live births)	(4) Under-5 mortality rate (per 1,000 live births)
	Panel A: Controlling for HIPC relief support - 1st variable definition			
Gavi eligibility effect	12.19***	9.13***	-7.72***	-15.24***
	(2.90)	(2.77)	(2.34)	(4.13)
Observations	1,735	1,735	1,096	1,096
Number of countries	84	84	82	82
Adjusted R-squared	0.36	0.30	0.80	0.79
	Panel B: Controlling for HIPC relief support - 2nd variable definition			
Gavi eligibility effect	10.33***	7.51***	-5.69**	-10.34***
	(2.83)	(2.64)	(2.23)	(3.69)
Observations	1,735	1,735	1,096	1,096
Number of countries	84	84	82	82
Adjusted R-squared	0.37	0.31	0.80	0.79
Country fixed-effects	Yes	Yes	Yes	Yes
Year fixed-effects	Yes	Yes	Yes	Yes
Covariates	Yes	Yes	Yes	Yes

Robust standard errors in parentheses clustered at the country level. *** p<0.01, ** p<0.05, * p<0.1. Full sample, unweighted regressions. First definition of HIPC relief support: Dummy variable equal to 1 time between HIPC decision point and completion, and 0 otherwise. Second definition of HIPC relief support: Dummy variable equal to 1 after HIPC decision point, and 0 otherwise. Main data source: World Development Indicators. Sample period: 1995–2016). All regressions include PPP-adjusted GDP per capita (in log) and population (in log) unless otherwise stated. Columns 3 and 4: Additional covariates are: primary school enrolment, secondary school enrolment, urban population, and political rights.

Table 5: Falsification test: effect on diseases Gavi doesn't target

	(1)	(2)	(3)
Dependent variables:	HIV prevalence (% of pop. ages 15-49)	Malaria incidence (per 1,000 pop.at risk)	TB incidence (per 100,000 people)
Gavi disbursements (real US$ m)	0.001	0.074	0.278
	(0.003)	(0.220)	(0.230)
Global Fund disbursements (real US$ m)	-0.013***	-0.466	-0.407**
	(0.004)	(0.285)	(0.190)
Observations	1,592	266	1,402
Number of countries	73	67	83
Adjusted R-squared	0.209	0.455	0.163
Country fixed-effects	Yes	Yes	Yes
Year fixed-effects	Yes	Yes	Yes
Covariates	Yes	Yes	Yes

Robust standard errors in parentheses clustered at the country level. *** p<0.01, ** p<0.05, * p<0.1. Main data source: World Development Indicators. Sample period: 1995–2016. All regressions include PPP-adjusted GDP per capita (in log) and population (in log) unless otherwise stated.

Table 6: DHS data results

Dependent variables:	(1) % of children 12-23 months who had received no vaccinations	(2) % of children 12-23 months who had received DPT3 vaccination	(3) % of children 12-23 months who had received Measles vaccination
Gavi eligibility effect	-5.13***	8.23***	11.16***
	(1.66)	(2.88)	(3.00)
Observations	146	146	146
Number of countries	54	54	54
Adjusted R-squared	0.39	0.60	0.59
Country fixed-effects	Yes	Yes	Yes
Year fixed-effects	Yes	Yes	Yes
Covariates	Yes	Yes	Yes

Robust standard errors in parentheses clustered at the country level. *** p<0.01, ** p<0.05, * p<0.1. Full sample, unweighted regressions. Data sources: Demographic and Health Surveys (outcome variables) and World Development Indicators (control variables). Sample period: 1995–2016. All regressions include PPP-adjusted GDP per capita (in log) and population (in log) unless otherwise stated.

Table 7: RDD-defined samples

Dependent variables:	(1)<br>Immunization, DPT<br>(% 12-23 months old)	(2)<br>Immunization, measles<br>(% 12-23 months old)	(3)<br>Infant mortality rate<br>(per 1000 live births)	(4)<br>Under-5 mortality rate<br>(per 1000 live births)
	Panel A. Narrow bandwidth sample: 0.5 log points of GNI on either side of Gavi eligibility cut-off			
Gavi eligibility effect	5.16**	3.97*	-2.66*	-5.85**
	(2.27)	(2.14)	(1.34)	(2.26)
Observations	1,276	1,276	774	774
Number of countries	61	61	59	59
Adjusted R-squared	0.21	0.19	0.82	0.79
	Panel B. Medium bandwidth sample: 1 log point of GNI on either side of Gavi eligibility cut-off			
Gavi eligibility effect	5.24***	4.59***	-3.17***	-6.85***
	(1.84)	(1.67)	(1.12)	(1.95)
Observations	1,979	1,979	1,202	1,202
Number of countries	95	95	92	92
Adjusted R-squared	0.27	0.23	0.80	0.77
Country fixed-effects	Yes	Yes	Yes	Yes
Year fixed-effects	Yes	Yes	Yes	Yes
Covariates	Yes	Yes	Yes	Yes

Robust standard errors in parentheses clustered at the country level. *** p<0.01, ** p<0.05, * p<0.1. Unweighted regressions. Main data source: World Development Indicators (control variables). Sample period: 1995–2016. All regressions include PPP-adjusted GDP per capita (in log) and population (in log) unless otherwise stated. Columns 3 and 4: Additional covariates are: primary school enrolment, secondary school enrolment, urban population, and political rights.

Table 8: Heterogeneous time effects

Dependent variables:	(1)<br>Immunization – DPT<br>(% 12-23 months old)	(2)<br>Immunization – measles<br>(% 12-23 months old)
Gavi eligibility effect – phase I	9.09***	5.74**
	(2.45)	(2.38)
Gavi eligibility effect – phase II	15.86***	12.68***
	(3.22)	(3.07)
Gavi eligibility effect – phase III	19.81***	15.80***
	(4.12)	(3.71)
Observations	333	333
Number of countries	84	84
Adjusted R-squared	0.52	0.46
Country fixed-effects	Yes	Yes
Year fixed-effects	Yes	Yes
Covariates	Yes	Yes

Robust standard errors in parentheses clustered at the country level. *** p<0.01, ** p<0.05, * p<0.1. Main data source: World Development Indicators. Sample period: 1995–2016. All regressions include PPP-adjusted GDP per capita (in log) and population (in log) unless otherwise stated.

Jaupart P, *et al. BMJ Global Health* 2019; 4:e001789. doi: 10.1136/bmjgh-2019-001789

Table 9: Effect of Gavi disbursements by programme type

	(1)	(2)	(3)	(4)	(5)	(6)	(7)	(8)	(9)	(10)	(11)	(12)
Dependent variables:	Immunization - DPT (% 12-23 months old)			Immunization - measles (% 12-23 months old)			Infant mortality rate (per 1000 live births)			Under-5 mortality rate (per 1000 live births)		
Gavi disbursements - System support (HSS, INS, and ISS)	0.34***			0.31***			-0.10*			-0.18		
	(0.09)			(0.08)			(0.06)			(0.11)		
Gavi disbursements - Vaccine support (NVS)		0.17**			0.13*			-0.10**			-0.18*	
		(0.07)			(0.08)			(0.05)			(0.10)	
Gavi disbursements - Total			0.15***			0.12**			-0.08**			-0.14*
			(0.05)			(0.06)			(0.04)			(0.08)
Observations	1,735	1,735	1,735	1,735	1,735	1,735	1,823	1,823	1,823	1,823	1,823	1,823
Number of countries	84	84	84	84	84	84	84	84	84	84	84	84
Adjusted R-squared	0.32	0.33	0.34	0.28	0.28	0.29	0.77	0.77	0.77	0.74	0.74	0.74
Country fixed-effects	Yes	Yes	Yes	Yes	Yes	Yes	Yes	Yes	Yes	Yes	Yes	Yes
Year fixed-effects	Yes	Yes	Yes	Yes	Yes	Yes	Yes	Yes	Yes	Yes	Yes	Yes
Covariates	Yes	Yes	Yes	Yes	Yes	Yes	Yes	Yes	Yes	Yes	Yes	Yes

Robust standard errors in parentheses clustered at the country level. *** p<0.01, ** p<0.05, * p<0.1. Main data source: World Development Indicators and Gavi's own disbursement portfolio . Sample period: 1995–2016. All regressions include PPP-adjusted GDP per capita (in log), population (in log). Gavi disbursements expressed in real US$ million. System support refers to the sum of Health system and immunisation strengthening (HSS), Injection safety support (INS), and (Immunisation services support). Vaccine support disbursements refer to New and underused vaccine support (NVS).

Jaupart P, *et al. BMJ Global Health* 2019; 4:e001789. doi: 10.1136/bmjgh-2019-001789

Figure 1: Late graduate countries – DPT immunization rates over time

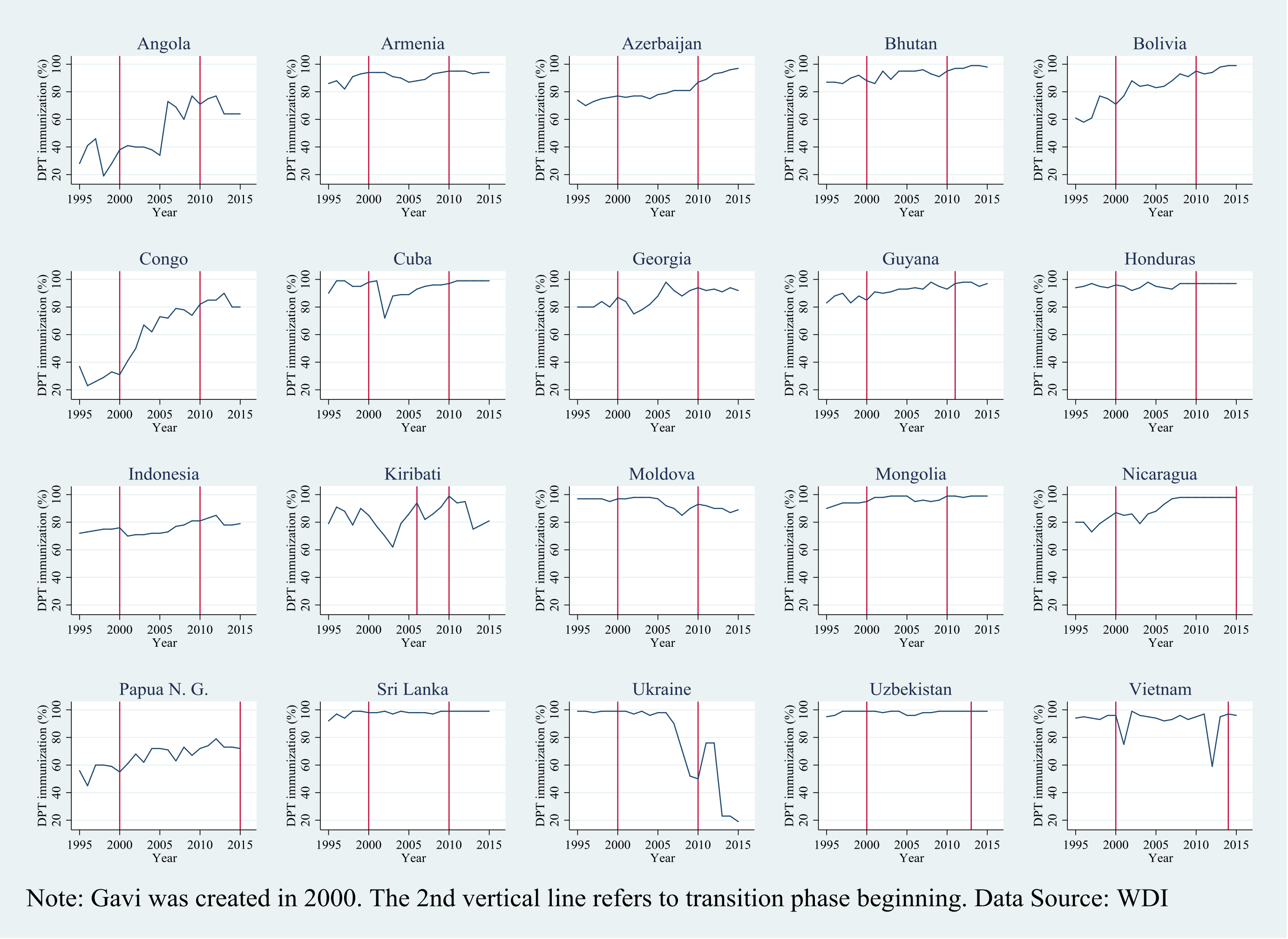

Jaupart P, *et al. BMJ Global Health* 2019; 4:e001789. doi: 10.1136/bmjgh-2019-001789

Table 10: Gavi effect on immunization rates, alternative samples and time periods

	(1)<br>Excluding early & late graduates, to 2016	(2)<br>Excluding early graduates only, to 2016	(3)<br>Excluding early & late graduates, to 2010	(4)<br>Excluding early graduates only, to 2010
	Panel A: Dependent variable – Immunization rate, DPT (% 12–23 months old)			
Gavi eligibility effect	12.02***	9.18***	8.06***	6.99***
	(2.75)	(2.61)	(2.25)	(1.96)
Observations	1,735	2,134	1,319	1,623
Number of countries	84	103	84	103
Adjusted R-squared	0.36	0.33	0.36	0.35
	Panel B: Dependent variable – Immunization rate, measles (% 12–23 months old)			
Gavi eligibility effect	8.81***	8.09***	5.55**	6.00***
	(2.63)	(2.38)	(2.30)	(2.01)
Observations	1,735	2,134	1,319	1,623
Number of countries	84	103	84	103
Adjusted R-squared	0.30	0.28	0.28	0.26
Country fixed-effects	Yes	Yes	Yes	Yes
Year fixed-effects	Yes	Yes	Yes	Yes
Covariates	Yes	Yes	Yes	Yes

Robust standard errors in parentheses clustered at the country level. *** p<0.01, ** p<0.05, * p<0.1. Main data source: World Development Indicators. Sample period: 1995–2016 (columns 1 and 2), 1995–2010 (columns 3 and 4). All regressions include PPP-adjusted GDP per capita (in log) and population (in log) unless otherwise stated.
